# Fractional crystallisation of eclogite during the birth of a Hawaiian Volcano

**DOI:** 10.1038/s41467-022-30108-x

**Published:** 2022-05-26

**Authors:** Laura A. Miller, Hugh St. C. O’Neill, Andrew J. Berry, Charles Le Losq

**Affiliations:** 1 School of Earth, Atmosphere and Environment, Monash University, Clayton, VIC 3800 Australia; 2 Research School of Earth Sciences, Australian National University, Canberra, ACT 2601 Australia; 3 Université Paris Cité, Institut de Physique du Globe de Paris, CNRS, F-75005 Paris, France

**Keywords:** Geochemistry, Petrology

## Abstract

The initial melts erupted by a Hawaiian volcano have a range of alkalic compositions but are rarely observed as they are covered by enormous volumes of shield stage tholeiites. A remarkable record of the early evolution of Hawaiian volcanoes, however, is preserved by a volcanic sandstone dredged from the submarine flank of Kilauea, which contains a suite of petrogenetically related pre-shield basanite to nephelinite glasses. Here we show that the systematic variation in the rare earth element (REE) patterns of these samples requires the fractional crystallisation of garnet. A fractionating assemblage of Ca-rich garnet (32%), omphacitic clinopyroxene (63%), and minor phlogopite can explain the variation in the major and trace element contents of the suite. The results suggest fractional crystallisation of eclogite from a primitive Hawaiian melt near the base of the lithosphere (>90 km) and that a deep magma chamber is the first stage in the development of a Hawaiian volcano.

## Abstract (web summary)

Rare glasses of earliest melts produced by a Hawaiian volcano can only have formed by the crystallisation of garnet implying a magma chamber near the base of the lithosphere

## Introduction

The birth of a Hawaiian volcano is an enigmatic stage in its growth. The lifecycle of an oceanic intraplate volcano is thought to evolve from an alkalic pre-shield stage with a moderately deep magma chamber (<30 km), to a tholeiitic shield-stage with a shallow magma chamber (<3 km) that produces 95% of the erupted products, before a return to alkalic magmatism in a post-shield stage^1–4^. The degree of melting varies throughout these stages with low degrees of melting at the edge of the plume generating the low SiO_2_, alkalic melts^5^. The youngest Hawaiian volcano, Loihi, is already entering shield-stage volcanism, while the pre-shield products of older Hawaiian volcanoes have been covered by vast amounts of tholeiitic shield-stage basalt. As a result, very little is known about the inception of Hawaiian volcanism with the scarcity of samples limiting our understanding of the low-degree melts generated during this early stage.

**Fig. 1:**
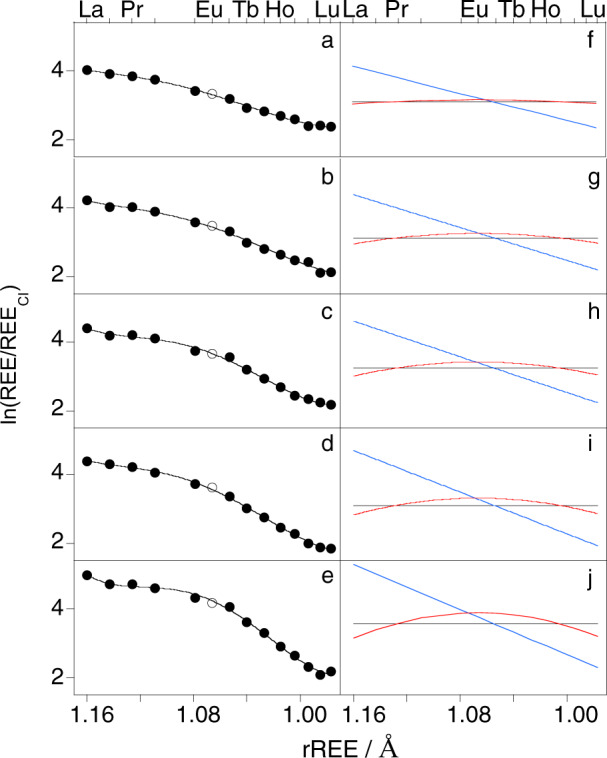
Fits of the rare earth element (REE) patterns of the basanite-nephelinite suite to orthogonal polynomials. The chondrite normalised abundances of the REE (symbols) in **a** the least evolved glass (B11), **b** C13, **c** C18, **d** B20, and **e** the most evolved glass (C4), and the fits (Eq.1; black line) to the data resulting from the sum of the orthogonal polynomials shown in **f**–**j** where the value of the average REE abundance (λ_0_; black) has been added to the orthogonal polynomials for slope (λ_1_f_1_; blue) and curvature (λ_2_f_2_; red) for ease of plotting. Eu was not included in the fits and is indicated by an open symbol in (**a**–**e**).

Volcaniclastic sandstones dredged from the south-eastern flank of Kilauea (site S508; Fig. 1 in Coombs et al.^6^) contain hyaloclastic spherules (Fig. S1) with a wide range of melt compositions, many of which are attributed to the pre-shield stage of Kilauea^3,7^. Among these glasses, are a set of 22 samples that exhibit a smooth continuous change in composition from basanite (sample B11) to nephelinite (sample C4), and progressive enrichment and depletion of incompatible and compatible elements, respectively (Tables S1 and S2; see group 1 glasses in Fig. 1 and Fig. 2 of Sisson et al.^8^). The glasses have trace element ratios that are distinct from prominent geochemical reservoirs (Table S3). For example, the average Ba/Th (ppm) is 350 ± 30, which exceeds that of both the bulk silicate Earth and ocean floor basalts (~80^9,10^), and typical Hawaiian tholeiites (80–200^11^). Further, many ratios of the most incompatible trace elements are essentially identical in the least and most evolved members of the suite (Table S3). The smooth change in major and trace element compositions and constant trace element ratios indicate that a single process formed the suite of glasses, for example, fractional crystallisation of an evolving melt, partial melting of a common source, or mixing of two melts that originated from the same source. That these glasses are a rare example of pre-shield magmatism and are petrogenetically linked endows them with considerable value.

Here, we address the petrogenesis of the suite of pre-shield basanite–nephelinite glasses from Kilauea by modelling the evolution of the REE patterns and the variations in trace element and major element compositions, for partial melting, fractional crystallisation, reactive melt flow and binary mixing. The changes in the REE patterns can only be reproduced by the fractional crystallisation of garnet, while the changes in trace and major element concentrations are consistent with the fractionation of eclogite. This implies the presence of a deep magma chamber during the inception of a Hawaiian volcano.

## Results and discussion

### The petrogenesis of Hawaiian pre-shield melts

**Fig. 2:**
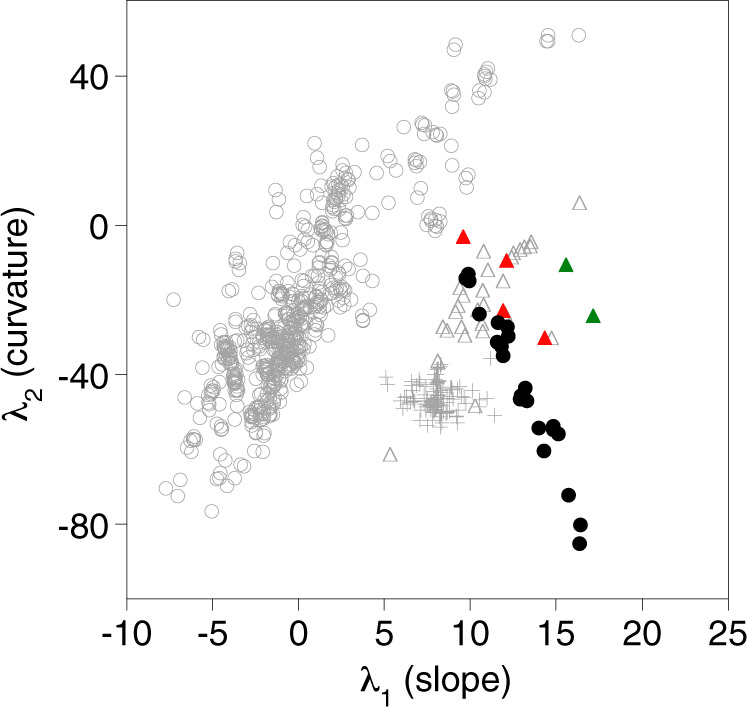
The curvature (λ_2_) and slope (λ_1_) of the rare earth element (REE) patterns of basaltic melts. The λ_2_ and λ_1_ of the REE patterns of ocean floor basalts (open circles^10^), Hawaiian shield tholeiites (crosses^13^), melts from Loihi (triangles^14,15^, including alkalic basalts (red) and basanites (green) for which fractionation of garnet has been suggested^15^), and the pre-shield Kilauean basanite-nephelinite glasses of this study (black).

To investigate the petrogenesis of the suite of glasses the REE patterns (Fig. S2) were fit to orthogonal polynomials using the method of O’Neill et al.^12^ (Fig. 1). The coefficients of the polynomials, λ_*n*_ (where λ_*n*_ = λ_0_, λ_1_ and λ_2_), parameterise the shape of a REE pattern in terms of the average REE abundance (λ_0_), slope (λ_1_) and curvature (λ_2_). This approach enables subtle differences in patterns to be distinguished and quantified. For example, if curvature is plotted against slope, ocean floor basalts, Hawaiian shield tholeiites and melts from Loihi occupy well-defined characteristic fields, whereas the glasses of the basanite-nephelinite suite define a remarkable linear trend (Fig. 2). The λ_*n*_ of the glass REE patterns are given in Table S4.

**Fig. 3:**
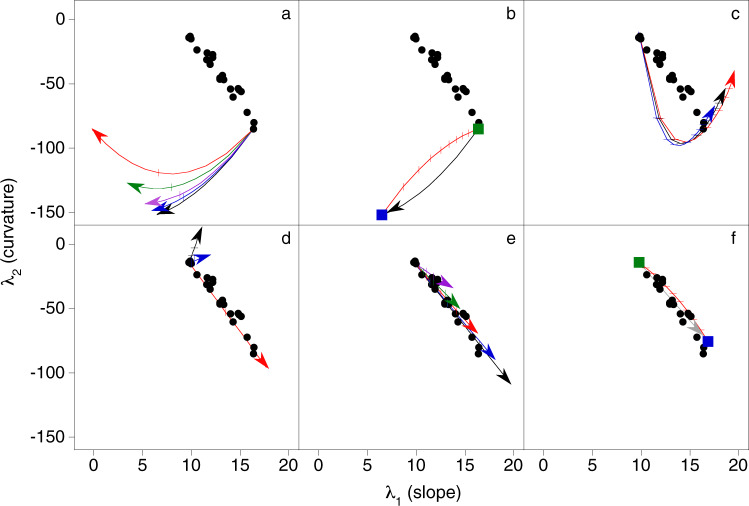
Modelled trends in the curvature (λ_2_) and slope (λ_1_) of rare earth element (REE) patterns due to partial melting, fractional crystallisation, reactive melt flow and binary mixing. The λ_2_ and λ_1_ and of the basanite-nephelinite glasses (black symbols) and trends in curvature and slope of the REE pattern of a melt due to **a** partial melting starting at the λ_1_ λ_2_ of the most evolved sample for 10% total melting (5% interval marked) where the relative proportions of clinopyroxene, orthopyroxene and olivine contributing to the melt are constant but the amount of garnet varies from 2 (black), 7 (blue), 14 (purple), 28 (green) to 50 (red) %, **b** linear mixing (red) of the most evolved sample (green square) and the melt (blue square) in (**a**) produced by 10% partial melting with 2% garnet (black vector), intervals of 10% are marked, **c** reactive flow of the least evolved melt flowing through a matrix of garnet peridotite with constant porosity (0.05; black), decreasing porosity (0.05–0.01; red) and increasing porosity (0.05–0.1; blue), intervals corresponding to a flow distance of 3, 7, 10% and then 10% increments to 80% are marked, **d** fractional crystallisation of 20% of garnet (red; i.e., 80% melt remaining), 40% clinopyroxene (blue) and 40% amphibole (black) from the least evolved sample, 10% intervals of crystallisation are marked, **e** fractional crystallisation of 40% garnet (gt) and clinopyroxene (cpx; i.e., 60% melt remaining) from the least evolved sample, where the fractionating assemblage varies from gt:cpx 0.5:0.5 (black), 0.4:0.6 (blue), 0.3:0.7 (red), 0.2:0.8 (green) to 0.1:0.9 (purple), 10% intervals of crystallisation are marked, and **f** linear mixing (red) of the least evolved sample (green square) and the residual melt (blue square) produced after that sample has undergone 42% fractional crystallisation of an assemblage consisting of 32% garnet 68% clinopyroxene (grey vector), 10% intervals of mixing are marked. Arrows show the direction of the petrogenetic processes.

Trends exhibited by the slope and curvature of REE patterns, such as that of the pre-shield basanite-nephelinite suite, can be modelled using the standard equations for partial melting and fractional crystallisation (e.g., Shaw^16^) and literature partition coefficients for common mantle minerals (garnet, clinopyroxene, orthopyroxene and olivine^12^). As garnet fractionates the REE more strongly than other mantle minerals it exerts the greatest control on the shape of a REE pattern and a range of models were produced by varying the amount of garnet melting or crystallising. The modelled slope and curvature trends are shown in Fig. 3, while the modelled average REE abundances, which are not as sensitive to different petrogenetic processes, are shown in Fig. S3. Partial melting was modelled to start at the slope and curvature of the most evolved sample and different models were produced by varying the amount of garnet contributing to the melt from 2 to 50% (Fig. 3a). The relative proportions of other contributing minerals were kept constant. Fractional crystallisation was modelled to start at the slope and curvature of the least evolved sample and different models were produced by varying the amount of garnet crystallising from 10 to 50% (Fig. 3d, e). Although, the proportion of garnet melting or crystallising is in some cases unrealistic they were modelled to illustrate the effect of garnet on REE pattern shape. Reactive melt flow, where a melt reacts with the matrix through which it is passing, can produce large enrichments in incompatible trace elements, similar to those exhibited by the present samples, and was also considered^17^. Models were produced for constant (0.05), increasing (0.05–0.1), and decreasing (0.05–0.01) matrix porosity with no mineralogical reaction, and for constant porosity and a mineralogical reaction of garnet peridotite to pyroxenite. The slope and curvature of the REE pattern of the first melt to flow through the matrix (i.e., the melt front) is shown in Fig. 3c and of subsequent melt batches in Fig. S4. The melt front modelled for the mineralogical reaction is the same as the model for constant porosity as melt flow is required for the reaction and a difference between these models is only observed in subsequent melt batches (Fig. S4). All the models are described in detail in the Methods section. Significantly, fractional crystallisation, partial melting and reactive melt transport result in very different λ_*n*_, with curved trends in slope and curvature for partial melting and reactive melt transport, though in opposing directions (Fig. 3a, c), and linear trends for fractional crystallisation (Fig. 3d, e). The REE patterns that correspond to these changes in slope and curvature are shown in Fig. S5.

At the low degrees of partial melting considered to produce alkalic melts (<10%^18^) the modelled trends proceed in a direction perpendicular to that defined by the slope and curvature of the glasses (Fig. 3a). Two co-genetic end-member melts formed by different degrees of partial melting would be connected by a partial melting trend such that binary mixing of these components would form a linear trend with a direction similar to that of the partial melting vectors (Fig. 3b). Thus, neither partial melting nor mixing of two end-member melts produced by partial melting of a common source can explain the observed changes in slope and curvature of the REE patterns.

Reactive melt flow produces curved trends in slope and curvature that also do not match the linear trend defined by the glasses (Fig. 3c; Fig. S4). The change in slope and curvature of the melt fronts suggest reactive melt flow could produce a melt (e.g., at a flow distance of 30% for a model of constant porosity and no mineralogical reaction) that could mix with the original melt to form the series. However, the almost exponential increase predicted for incompatible trace elements (e.g., Fig. S3c) is not seen in the glasses, while it would also seem fortuitous that a melt produced at this particular flow distance formed binary mixing trend. Thus, as for partial melting, neither reactive melt flow nor binary mixing of an initial melt and a component produced by reactive melt flow can explain the changes in the REE patterns of the glasses.

The linear slope and curvature trend for the fractional crystallisation of garnet is identical to that of the glasses (Fig. 3d) suggesting that this process formed the melt suite. The crystallisation of garnet would decrease the average REE concentration of a melt (average REE garnet/melt partition coefficient, ${D}_{{{{{{\rm{REE}}}}}}}^{{{{{{\rm{garnet}}}}}}/{{{{{\rm{melt}}}}}}}$ > 1; Fig. S3b) and the relatively constant REE content of the glasses (λ_0_; Table S4) suggests that another phase(s) with ${{D}}_{{{{{{\rm{REE}}}}}}}^{{{{{{\rm{crystal}}}}}}/{{{{{\rm{melt}}}}}}}$ < 1 must be crystallising alongside garnet. The crystallisation of clinopyroxene, amphibole, orthopyroxene, olivine, and phlogopite would have a negligible effect, relative to that of garnet, on the slope and curvature of the REE pattern of a residual melt (Fig. 3d). Indeed, the low compatibility of the REE in orthopyroxene, olivine and phlogopite means that the trends due to the crystallisation of these minerals are too small to be shown in Fig. 3d. The trends produced by clinopyroxene and amphibole have very different orientations to that of garnet, however, the relative magnitudes of the vectors means that the vector sum of garnet and clinopyroxene and/or amphibole would have almost the same direction as the garnet trend, such that the crystallisation of garnet masks the presence of other crystallising phases (Fig. 3e). Hence, while the average REE abundance (λ_0_), slope (λ_1_), and curvature (λ_2_) of the glasses indicate that the suite was formed through fractional crystallisation involving garnet, little can be said about the other phase(s) also crystallising. Mixing of two cogenetic end-member melts that are related by fractional crystallisation involving garnet would produce a linear trend that is indistinguishable from the trend produced by garnet crystallisation (Fig. 3f). Although, in this case the petrogenetic implications of the two processes are the same as they both necessitate the crystallisation of garnet.

The minerals that would crystallise from the least evolved composition (B11) were determined by experiments from 3 to 5 GPa and 1300–1450 °C. Garnet was identified as a liquidus phase in the highest pressure experiment (Fig. S6), while clinopyroxene crystallised at all pressures (Table S5a). The garnet crystals have atomic Ca/(Ca+M) ~ 0.4, where M = Mg + Fe + Mn, which is typical of eclogitic^19^ and distinct from peridotitic garnets, where Ca/(Ca+M) is buffered to lower values by the presence of orthopyroxene (~ 0.13^20^). The jadeite component of the clinopyroxene is ~ 0.3, which is similar to that of the eclogitic pyroxene omphacite. Therefore, the experiments suggest that the least evolved composition would fractionate an assemblage with an eclogitic composition.

The generation of the suite was further explored by calculating the bulk crystal/melt partition coefficients (*D*_M_^crystal/melt^) of the trace elements (M). The two most evolved glasses were excluded from this calculation, and also from the derivation of the cotectic assemblage that follows, as they show marked increases or decreases in the concentrations of some elements relative to the other glasses of the suite (e.g., Ni, FeO; Tables S1, S2; see “Methods”). To determine *D*_M_^crystal/melt^ the trace element contents of the glasses were fit to the equation for fractional crystallisation by least squares regression (see “Methods”). The resulting *D*_M_^crystal/melt^ are given in Table S6, and the degrees of crystallisation required to produce each glass from the least evolved composition (B11), which is a subsidiary result of the calculation, are given in Table S4. The glasses are related by a total of 40% fractional crystallisation (i.e., 60% residual melt), while the *D*_M_^crystal/melt^ are generally consistent with a fractionating assemblage of garnet and clinopyroxene^21^ (Fig. S7). The high CO_2_ contents of the glasses (Table S1) indicate that they were erupted at a water-column pressure sufficient to prevent complete degassing of CO_2_. As CO_2_ is preferentially fractionated into vapour with respect to H_2_O^22^ the relatively high partition coefficient of H_2_O (0.57; Table S6) can be attributed to moderately compatible behaviour rather than degassing. This suggests that a hydrous phase such as phlogopite or pargasite was also part of the fractionating assemblage. Phlogopite is stable at higher pressures than pargasite^23,24^, which in addition to the moderately compatible behaviour of Rb and Ba (Table S6^25^), indicates that phlogopite is more likely. Phlogopite was not crystallised experimentally since the experiments were nominally anhydrous. Cr has a particularly high partition coefficient suggesting Cr-rich garnet or clinopyroxene.

**Fig. 4:**
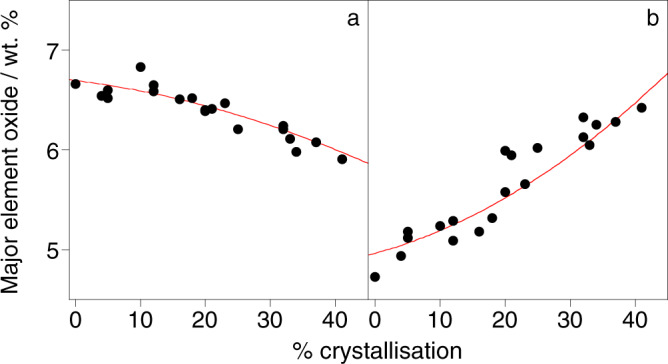
Major element contents of the basanite-nephelinite suite. Measured (black) and predicted (for a fractionating assemblage of clinopyroxene (63%), garnet (32%), and phlogopite (5%); red) **a** MgO and **b** Na_2_O contents of the glasses versus the degree of crystallisation determined from fitting the trace element contents to the equation for fractional crystallisation (Eq. 12).

The REE pattern shape coefficients and trace element bulk partition coefficients indicate that the suite was formed by the crystallisation of garnet, clinopyroxene and phlogopite. Thus, the changes in major element concentrations, which represent the bulk fractionating composition, were decomposed into these mineral components using least squares regression (see “Methods”). The best fit to the composition was an assemblage of clinopyroxene (63%), garnet (32%), and phlogopite (5%), using a stoichiometric composition for phlogopite and the experimentally determined compositions of clinopyroxene and garnet (Table S5b). The agreement between the measured and modelled major element compositions is illustrated for MgO and Na_2_O, as a function of the degree of crystallisation determined from the trace element data, in Fig. 4.

The consistency between the REE shape modelling, the compositions determined experimentally, the modelled partition coefficients, and the modelled cotectic assemblage identify fractional crystallisation as the process by which the suite of glasses was formed. An alternative method of parameterising REE shape, using Dy/Yb to approximate the slope and Dy/Dy*, where Dy* is the value for Dy obtained by linear interpolation between La and Yb, to approximate the curvature, similarly indicates that the glass suite was formed through the fractional crystallisation of garnet (Fig. S8; see Fig. 4d in Davidson et al.^26^). The proportion of garnet crystallising (32% of the fractionating assemblage) and the degree of crystallisation (40%) were determined by models independent of the REE shape modelling. Yet the predicted change in REE pattern slope and curvature for 40% crystallisation with garnet forming ~32% of the fractionating assemblage is an extraordinarily close match to the trend in slope and curvature of the suite of glasses (red trend; Fig. 3e; with the two most evolved glasses excluded as these were not included in the modelling).

### Fractional crystallisation beneath pre-shield Kilauea

The fractionation of garnet from primitive mantle melts was shown to produce alkalic melts by early experimental studies^27,28^. However, this process fell out of favour as later work showed that varying degrees of partial melting could generate the range of alkalic melts seen at Hawaii^29^. Indeed, the basanite-nephelinite suite of the present study was attributed to varying degrees of partial melting of a garnet lherzolite source^8^. However, modelling of the REE patterns unambiguously shows that the suite was formed by fractional crystallisation rather than partial melting (Fig. 3). More recently, it has been shown that orthopyroxene and garnet can crystallise at the expense of olivine from a carbonated silicate melt to produce nephelinite and basanite compositions^30^. The fractional crystallisation of eclogite from alkalic melts has also been proposed previously^31^ but not, to our knowledge, for Hawaiian pre-shield melts.

The development of relatively deep (<30 km) ephemeral magma chambers in the early stages of intraplate oceanic volcanoes is well documented^2,32–34^, where low melt flux results in deep storage^35^. Melts erupted by these young volcanoes can be the products of extensive fractionation as they stall in the cold lithosphere before establishing an open conduit to the surface. The formation of the pre-shield suite of glasses by fractional crystallisation suggests that a magma chamber (this term is used here without implications for size, shape or longevity) similarly formed during the birth of Kilauea. Garnet occurs on the liquidus of the least evolved composition at 5 GPa (~150 km), however, as the experiments may not exactly replicate natural conditions these results do not necessarily suggest a magma chamber at 150 km. There is a strong association between alkalic melts and carbonated sources^36^ and the presence of CO_2_ increases the stability field of garnet, with garnet being present on the liquidus of carbonated basanite/nephelinite (>2.6 wt.% CO_2_) melts at pressures as low as 3 GPa (90 km^30^). The CO_2_ contents of the present glasses (up to 0.8 wt.%; Table S1) may not be representative of their original contents as they could have experienced degassing. Seismic studies indicate that the lithosphere beneath Hawaii extends to ~110 km^37^ and has some rigidity to ~150 km^38^, while the thickness of old oceanic lithosphere, which the leading edge of the Hawaiian hotspot would encounter, is ~100 km^37,38^. Melt is considered to be generated at depths 90–120 km beneath Kileaua^4,39^ with earthquake foci tracking magma transport from depths > 50 km^40,41^. Rare garnet pyroxenite xenoliths erupted by rejuvenated volcanism at Oahu are considered to be accumulates that originated at depths between 60 and 90 km (Bizimis et al.^42^ and references therein), further indicating stable lithosphere deep beneath Hawaii.

The seismic and petrological evidence suggest that a deep magma chamber is possible, with the stability of garnet in the experiments of Mallik and Dasgupta^30^ placing an upper limit on the depth. This depth (>90 km) is, however, strikingly deeper than that invoked for other oceanic intraplate volcanoes (< 30 km^34^). Hawaii has often been considered the archetype of hotspot volcanism, although as the buoyancy flux is at least twice that of others^43^ (apart from Iceland) it is more likely to be the exception. The development of an unusually deep pre-shield magma chamber is therefore perhaps in keeping with the atypical character of Hawaii. Alkalic pre-shield melts at Loihi that are thought to have been differentiated by the fractionation of garnet^15^ show a similar linear trend between the curvature and slope of the REE patterns as observed for the glasses studied here (Fig. 2). This suggests that deep fractional crystallisation may have also played a role in the genesis of melts erupted by Loihi and could be a general process for young Hawaiian volcanoes.

Both fractional crystallisation and partial melting play important roles in the generation of alkalic melts. Here we demonstrate unambiguously the role of garnet crystallisation in the formation of pre-shield stage Hawaiian melts. This challenges the viewpoint that fractional crystallisation is solely a shallow process and suggests that the development of a deep (> 90 km) magma chamber is an important early stage in the birth of a Hawaiian volcano.

## Methods

### Experimental

A synthetic version of the least evolved glass (B11) was prepared using powders of reagent grade oxides, carbonates, and synthetic fayalite. The oxide and carbonate powders were dried prior to weighing. All of the components were mixed under acetone using an agate pestle and mortar for ~20 min. The mixture was pressed into a pellet and de-carbonated at 1000 °C for > 12 h. The pellet was then crushed and ground for ~30 min under acetone. The resulting powder was equilibrated over a range of temperatures (1300–1450 °C) and pressures (3–5 GPa) for 24 h, in graphite capsules, using MgO-pyrex-NaCl assemblies, and an end-loaded piston-cylinder apparatus. Each capsule was mounted in epoxy resin and ground back to expose the sample, which was then polished.

### Analytical

A JEOL 8530F electron probe microanalyser (EPMA) operating at 15 kV and 80 nA, with a spot size of 10 μm, was used to determine the major element compositions of the natural glasses. The EPMA was calibrated using Astimex Standards Ltd mineral standards and VG2 for S. The major element compositions determined in this study and in Sisson et al.^8^ agree within 3%, other than Na_2_O and MnO, which are within 6 and 8%, respectively. There is a large difference between the S contents of the two studies, which is attributed to differences in the S content assumed for VG2 (1200 ± 80 ppm in Coombs et al.^44^ and 1420 ppm here; see O’Neill^45^ for a compilation). The natural glasses and experimental samples were examined using a JEOL 6400 scanning electron microscope operating at 15 kV. Back-scattered electron (BSE) images were used to determine the proportion of phases in the experimental samples by counting the pixels of the grey-scale associated with each phase (e.g., the garnet crystals are pale grey; Fig. S6) and dividing by the total pixels in the image. The compositions of the synthetic glasses and crystals were determined using energy dispersive spectroscopy. The phase equilibrium assemblages and the average compositions of the crystals are given in Tables S5a and S5b.

Attenuated total reflectance Fourier transform infra-red (ATR-FTIR) spectra were recorded from the natural glasses using a Ge ATR crystal with a refractive index of 4.0, a Hyperion infrared microscope, and a Bruker Tensor 27 spectrometer equipped with a KBr beamsplitter and liquid N_2_ cooled DTGS detector. The chamber was purged with dry air prior to and during acquisition of the spectra and a background was recorded after every six spectra. The infra-red spot size was 10 µm and the penetration depth ~1 µm. Spectra were recorded from 650 to 5000 cm^−1^ with a spectral resolution of 4 cm^−1^. Synthetic mid-ocean ridge basalt (MORB) glasses with known H_2_O contents were used as standards^46^. Three replicate spectra were recorded for all samples and standards. For each spectrum the area of the O–H band between 3100 and 3750 cm^−1^ was normalised to that of the silicate band between 810 and 850 cm^−1^ and the H_2_O contents determined using a calibration based on the standards^47^. The average difference between the water contents determined for the standards and the published values was 0.11 wt.%. Low intensity peaks at 1410 and 1501 cm^−1^ observed in some of the spectra were assigned to vibrations of CO_3_^2^^−^ in the glasses^48^ and used to determine the concentration of CO_2_^49^. The H_2_O and CO_2_ contents of the glasses are given in Table S1. The CO_2_ contents have a logarithmic relationship with the total alkali content (Fig. S9), in contrast to the linear relationship predicted for CO_2_ solubility^22^, but in keeping with a reciprocal solution model proposed for S^2-^ ^45^. If this model is appropriate for CO_2_, the correlation suggests degassing at constant pressure with the different CO_2_ contents being controlled by composition.

The trace element concentrations of the natural glasses were determined by laser ablation inductively coupled plasma mass spectrometry, LA-ICP-MS, using analytical protocols and standards similar to those of Jenner and O’Neill^50^. The instrument comprises a 193 nm wavelength ArF EXCIMER laser coupled to an Agilent 7500S quadrupole ICP-MS. The diameter of the analysis spot was 28 µm. The acquisition time per analysis was 70 s, with the first and last 20 s recorded as background before and after ablation. Glass analyses were obtained in batches of up to nine, bracketed before and after by analyses of standards. The trace element concentrations were determined using two routines. The first routine comprised the elements reported by Sisson et al.^8^ (^29^Si, ^43^Ca, ^85^Rb, ^88^Sr, ^89^Y, ^90^Zr, ^93^Nb, ^138^Ba, the REE, ^177^Hf, ^181^Ta, ^207^Pb, ^232^Th, ^238^U) and was recorded for glasses not analysed in that study (B11, B10, B19, and B20). The second routine comprised ^7^Li, ^9^Be, ^29^Si, ^43^Ca, ^45^Sc, ^51^V, ^53^Cr, ^55^Mn, ^59^Co, ^60^Ni, ^65^Cu, ^66^Zn, ^71^Ga, ^95^Mo, ^118^Sn, and ^205^Tl, and was recorded for all of the glasses for which there was space for a laser spot. Replicate analyses were recorded where possible. NIST 612 was used as an external standard, and NIST 610 and BCR-2G as secondary standards. The Ca content of the glasses determined by EPMA and the count rate of ^43^Ca was used as the internal standard. The Python package LAtools was used for data reduction^9,51^. The LA-ICP-MS analyses of the standards have a precision, defined by the relative standard deviation, of < 5%, except for Tl, which is 10%. The LA-ICP-MS results from this study are given in Table S2, together with data from Sisson et al.^8^ for glasses where new analyses were not possible. There appears to be a systematic offset (~10%) between the results of the two studies based upon differences in the smooth trends between trace elements and total alkali content. This offset is attributed to differences in the standards or data reduction methods.

### λ_*n*_ fitting

The geochemical behaviour of most of the REE is controlled by the decrease in ionic radius with increasing atomic number for constant charge (3+). Consequently, the abundances of the REE in a melt vary as a function of their radii (r_REE_) and the resulting REE pattern can be fit to orthogonal polynomials that are derived from the radii^12^. The obvious exceptions to this are Ce and Eu, which can occur as Ce^4+^ and Eu^2+^, and these REE should be excluded from the fit if Ce^4+^ and/or Eu^2+^ are present. Orthogonal polynomials are used to ensure that the coefficients of the polynomials vary independently of each other, although this is only strictly true if the same number of REE are used in the fit as were used to derive the polynomials. The orthogonal polynomials used here (given in Tables 1 and 2 of O’Neill^12^) were derived from the radii of the same 13 REE fit in the present study (Eu was excluded as it commonly occurs as Eu^2+^ in basalts). The fit of the orthogonal polynomials to a REE pattern can be written as:

1 $${{{{\mathrm{ln}}}}}([{{{{{\rm{REE}}}}}}]/{[{{{{{\rm{REE}}}}}}]}_{{{{{{\rm{CI}}}}}}})={{{{{\uplambda }}}}}_{0}{{{{\rm{f}}}}_{0}}^{{{{{{\rm{orth}}}}}}}+{{{{{\uplambda }}}}}_{1}{{{{\rm{f}}}}_{1}}^{{{{{{\rm{orth}}}}}}}+{{{{{\uplambda }}}}}_{2}{{{{\rm{f}}}}_{2}}^{{{{{{\rm{orth}}}}}}}+{{{{{\uplambda }}}}}_{3}{{{{\rm{f}}}}_{3}}^{{{{{{\rm{orth}}}}}}}+{{{{{\uplambda }}}}}_{4}{{{{\rm{f}}}}_{4}}^{{{{{{\rm{orth}}}}}}}+{{{{{\rm{etc}}}}}}.,$$

where [REE]/[REE]_Cl_ refers to the chondrite (CI) normalised REE abundances, f_*n*_^orth^ to the orthogonal polynomials and λ_*n*_ to the coefficients of the orthogonal polynomials, where *n* is the order of the polynomial. Higher order functions than f_2_^orth^ commonly reflect nothing more than ‘noise’^12^. The manner in which the coefficients and polynomials describe the shape of a REE pattern is illustrated by example fits shown in Fig. 1. The orthogonal polynomials describe different shape components: f_0_^orth^ is a constant function (black line in Fig. 1f–j), f_1_^orth^ a linear function (blue line in Fig. 1f–j), and f_2_^orth^ a quadratic function (red line in Fig. 1f–j). The coefficients parameterise the contribution of each polynomial to the REE pattern shape: λ_0_ is the average REE concentration of the pattern (i.e., the *y* intercept of the constant function; λ_0_ = 3.10 in Fig. 1f and λ_0_ = 3.56 in Fig. 1j), λ_1_ the slope of the REE pattern (i.e., the gradient of the linear function; λ_1_ = 9.75 in Fig. 1f and λ_1_ = 16.34 in Fig. 1j), and λ_2_ the curvature of the REE pattern (i.e., the openness or tightness and upward or downward opening of the quadratic function; λ_2_ = −14.1 in Fig. 1f and λ_2_ = −85.1 in Fig. 1j). The shape coefficients, λ_*n*_, were calculated for the REE patterns of the glasses. The λ_*n*_ are given in Table S4_._

### λ_*n*_ modelling

The partition coefficients, *D*_M_^crystal/melt^ where M is an element, of the REE are smooth functions of r_REE_ and can be fit to the orthogonal polynomials in the same way as the REE patterns:

2 $${{{D}}_{{{{{{\rm{REE}}}}}}}}^{{{{{{\rm{crystal}}}}}}/{{{{{\rm{melt}}}}}}}={{{{{{\rm{\delta }}}}}}}_{0}{{{{\rm{f}}}}_{0}}^{{{{{{\rm{orth}}}}}}}+{{{{{{\rm{\delta }}}}}}}_{1}{{{{\rm{f}}}}_{1}}^{{{{{{\rm{orth}}}}}}}+{{{{{{\rm{\delta }}}}}}}_{2}{{{{\rm{f}}}}_{2}}^{{{{{{\rm{orth}}}}}}}+{{{{{{\rm{\delta }}}}}}}_{3}{{{{\rm{f}}}}_{3}}^{{{{{{\rm{orth}}}}}}}+{{{{{{\rm{\delta }}}}}}}_{4}{{{{\rm{f}}}}_{4}}^{{{{{{\rm{orth}}}}}}}+{{{{{\rm{etc}}}}}}.,$$

where the δ_*n*_ coefficients describe the shape of the REE partitioning pattern (in the same way as λ_*n*_ describes the shape of a REE pattern). The right hand side of Eq. 2 can then be substituted for ${D}_{{{{{{\rm{REE}}}}}}}^{{{{{{\rm{crystal}}}}}}/{{{{{\rm{melt}}}}}}}$ in models of petrogenetic processes.

Models of fractional crystallisation were constructed using the equation:^16^

3 $$[{{{\rm{M}}}}]/[{{{{\rm{M}}}}}_{0}]={{F}}^{{D}-1}$$

which rearranges to:

4 $${{{{\mathrm{ln}}}}}([{{{\rm{M}}}}]/[{{{{\rm{M}}}}}_{0}])=\,{{{{\mathrm{ln}}}}}({F})({{D}}_{{{{{{\rm{M}}}}}}}-1)$$

where [M] is the concentration of element M in the melt, [M_0_] the initial concentration, *F* the mass fraction of melt (which varies between 0 and 1, where 1 is zero crystallisation) and *D*_M_ the bulk partition coefficient, Σ*D*_M_^crystal/melt^*m*_*x*_, where *m*_*x*_ is the mass fraction of the crystalline phase *x*. Combining Eq. 2 with Eq. 4 gives:

5 $${{{\rm{\psi}}}}_{0}=\,{{{{\mathrm{ln}}}}}({F})\left(\sum {{m}}_{{x}}{{{{{\rm{\delta }}}}}}_{0}-1\right),{{{\rm{\psi}}}}_{1}=\,{{{{\mathrm{ln}}}}}({F})\left(\sum {{m}}_{{x}}{{{{{\rm{\delta }}}}}}_{1}\right)\,{{{{{\rm{and}}}}}}\,{{{\rm{\psi}}}}_{2}=\,{{{{\mathrm{ln}}}}}({F})\left(\sum {{m}}_{{x}}{{{{{\rm{\delta}}}}}}_{2}\right)$$

where Ψ_0_, Ψ_1_, and Ψ_2_ are termed petrogenetic process vectors, which describe the change in average REE abundance (Ψ_0_), slope (Ψ_1_) and curvature (Ψ_2_) between the initial and observed REE patterns. The change in REE pattern shape can also be determined by using Eq. 3 to generate multiple REE patterns as a function of the degree of crystallisation (*F*). These REE patterns could then be fit to the orthogonal polynomials to quantify the differences between patterns, or change in λ_*n*_ (Ψ_*n*_), due to fractional crystallisation. However, this method is time consuming as it requires fitting many REE patterns to the orthogonal polynomials rather than a single fit to the partition coefficients.

The partial melting models were constructed using the equation for batch melting:^16^

6 $$[{{{\rm{M}}}}]/\left[{{{{\rm{M}}}}}_{0}\right]=1/\left[{D}_{0}+{F}(1-{P})\right]$$

or

7 $${{{{\mathrm{ln}}}}}\left([{{{\rm{M}}}}]/\left[{{{\rm{M}}}}_{0}\right]\right)=-{{{{\mathrm{ln}}}}}\left({D}_{0}-{FP}+{F}\right)$$

where *F* is the mass fraction of melt, *D*_0_ the initial bulk partition coefficient given by Σ*D*_M_^crystal/melt^*m*_*x*_^0^, where *m*_*x*_^0^ is the mass fraction of the crystalline phase *x* in the source, and *P* the bulk distribution coefficient for the melting assemblage of minerals given by Σ*D*_M_^crystal/melt^*p*_*x*_, where *p*_*x*_ is the mass fraction of the crystalline phase *x* entering the melt. It is not possible to construct simple petrogenetic process vectors (Ψ_*n*_) for this equation. As a result Eq. 6 was used to determine the amounts of each REE, and hence the REE patterns, as a function of *F*, which were then fit to the orthogonal polynomials (Eq. 1). The change in pattern shape as a function of *F* could then be determined.

The effects of fractional crystallisation and partial melting on REE pattern shape were modelled using the methodology described above (worked examples of both processes are included as an excel spreadsheet in the supplementary information; Worked Example 1). The shapes of the REE mineral/melt partition coefficient patterns (as parameterised by δ_*n*_) used in the models are from Table 7 in O’Neill^12^. It is the relative difference between the *D*_REE_^crystal/melt^ (i.e., the pattern shape) that is important for defining the direction of a process in lambda space (e.g., the fractional crystallisation vectors in Fig. 3d), with the absolute values of *D* defining the magnitude of the change (e.g., the length of the vectors in Fig. 3d). This means that vectors for the different mantle minerals have very different directions (Fig. 3d), but the vectors for a particular mineral (generated using different ${D}_{{{{{{\rm{REE}}}}}}}^{{{{{{\rm{crystal}}}}}}/{{{{{\rm{melt}}}}}}}$) all have a similar direction. Thus, the values of ${D}_{{{{{{\rm{REE}}}}}}}^{{{{{{\rm{crystal}}}}}}/{{{{{\rm{melt}}}}}}}$ used in the models, within the typical ranges, make little difference to the direction of the mineral vectors.

Fractional crystallisation was modelled to start from the least evolved glass (B11; Fig. 3d,e), and partial melting from the most evolved glass (C4; Fig. 3a). In both cases, these are melt compositions rather than source compositions. These starting points were chosen to facilitate direct comparison with the basanite-nephelinite λ_*n*_. The proportion of garnet melting (Fig. 3a) and crystallising (Fig. 3e) was varied in the models. Relative to garnet and clinopyroxene, the REE have lower compatibilities in other common mantle minerals (i.e., orthopyroxene, olivine, and spinel) meaning that these minerals could be omitted from models of fractional crystallisation (Fig. 3e).

Reactive melt flow was modelled following the incremental method of Reiners^17^. In this approach the matrix or column through which the melt flows is divided into a series of cells, where it takes the melt one time step (*j*) to advance through one cell (*i*). The concentration of an element (C) is determined in each cell for each timestep for a sequence of cells (0 to *i*) and time steps (0 to *j*):

8 $${{{\rm{C}}}}_{{{{{\rm{f}}}}}_{{i}}}^{{j}}=\frac{{{{{{{\rm{\rho }}}}}}}_{{{{{{{\rm{f}}}}}}}}{{{{{{\rm{\varphi }}}}}}}_{{i}-1}^{{j}-1}{{{\rm{C}}}}_{{{{{{{\rm{f}}}}}}}_{i-1}}^{{j}-1}+{{{{{{\rm{\rho }}}}}}}_{{{{{{\rm{s}}}}}}}(1-{{{{{{\rm{\varphi }}}}}}}_{{i}}^{{j}-1}){{{\rm{C}}}}_{{{{{{{\rm{s}}}}}}}_{{i}}}^{{j}-1}}{{{{{{{\rm{\rho }}}}}}}_{{{{{{\rm{f}}}}}}}{{{{{{\rm{\varphi }}}}}}}_{{i}}^{{j}}+{{{{{{\rm{\rho }}}}}}}_{{{{{{\rm{s}}}}}}}(1-{{{{{{\rm{\varphi }}}}}}}_{{i}}^{{j}}){{D}}_{{{{{{\rm{M}}}}}}i}^{{j}}}$$

where C_f_ is the initial concentration of the element in the melt, ρ_f_ the density of the melt, C_s_ the initial concentration of the element in the matrix, ρ_s_ the density of the matrix, ${{{{{\rm{\varphi }}}}}}$ the porosity of the matrix and *D*_M_^crystal/melt^ the crystal melt partition coefficients. As the melt moves it remains in local equilibrium with the matrix such that:

9 $${{{\rm{C}}}}_{{{{{{\rm{s}}}}}}i}^{{j}}={{{\rm{C}}}}_{{{{{{\rm{f}}}}}}i}^{{j}}({{D}}_{{i}}^{{j}})$$

In models of changing porosity, from an initial value ${{{{{\rm{\varphi }}}}}}$_0_ to a final value ${{{{{\rm{\varphi }}}}}}$_1_, a rate of change (*r*) of 0.05 per cell was used. To conserve mass within a column ${{{{{\rm{\varphi }}}}}}$ was changed at a uniform rate for all cells at each time step such that ${{{{{\rm{\varphi }}}}}}$ = ${{{{{\rm{\varphi }}}}}}$_0_ when *j* = 0, and in subsequent time steps:

10 $${{{\rm{\varphi}}}}^{j}={{{\rm{\varphi}}}}^{{j}-1}+{r}\left({{{\rm{\varphi}}}}_{1}\right)-{{{\rm{\varphi}}}}^{{j}-1}$$

The change in partition coefficient due to a mineralogical reaction, from an initial value (*D*_M_^0^) for the original mineral assemblage (e.g., garnet peridotite) to a final value (*D*_M_^1^) for the resultant assemblage (e.g., pyroxenite), was modelled using the same rate of change. The *D*_M_ of the melt front is *D*_M_^0^ and for subsequent melt batches is given by:

11 $${{{D}}_{{{{{{\rm{M}}}}}}}}^{{j}}={{{D}}_{{{{{{\rm{M}}}}}}}}^{{j}-1}+{r}({{{D}}_{{{{{{\rm{M}}}}}}}}^{1}-{{{D}}_{{{{{{\rm{M}}}}}}}}^{{j}-1})$$

Reactive melt flow models used an input melt with the composition of the least evolved glass (B11), a garnet peridotite matrix, ρ_f_ = 2800 kg/m^3^, ρ_s_ = 3300 kg/m^3^ and ${{{{{\rm{\varphi }}}}}}$_0_ = 0.05. In models of decreasing and increasing porosity ${{{{{\rm{\varphi }}}}}}$_1_ was 0.01 and 0.1 respectively. In a model of changing matrix mineralogy *D*_M_ were changed from those of a garnet peridotite (*D*_M_^0^) to those of a pyroxenite (*D*_M_^1^), where the *D*_M_ were calculated from the mineral-melt partition coefficients for the reaction 65% olivine + 24% clinopyroxene + 6% orthopyroxene + 5% garnet = 10% olivine + 75% clinopyroxene + 15% garnet. The column through which the melt flows was divided into 30 cells and the concentration of each REE was calculated using Eq.8 to Eq. 11 for each cell for *i* = 0 to 30 and *j* = 0 to 120. Each cell was assigned a length of 100 m and the time taken for the melt to flow through a single cell was 20 y. The time taken for the melt to flow through one column (3000 m) is thus 600 y. The values of these parameters (ρ_f_, ρ_s_, ${{{{{\rm{\varphi }}}}}}$, ${{{{{\rm{\varphi }}}}}}$^1^, *D*_M_, *D*_M_^1^ cell size and melt flow rate) were taken from Reiners^17^. A worked example is included as an excel spreadsheet in the supplementary information (Worked Example 2). The REE patterns of the melt front and of subsequent melts at discrete time intervals were fit to orthogonal polynomials^12^ to parameterise the shape of the patterns. The change in REE pattern shape due to reactive melt flow was then determined.

### Trace element bulk crystal-melt partition coefficients and the degree of crystallisation

To determine the bulk partition coefficient (*D*_M_) of each element (M) and the degree of crystallisation (*F*) for each glass the concentration of each trace element in each sample (*n*_s_) was fit to the equation for fractional crystallisation (Eq. 3) in the form:

12 $${{{{\mathrm{ln}}}}}[{{{{\rm{M}}}}}_{({{{{{\rm{calc}}}}}})}]=[{{{{{{\rm{ln}}}}}}{{{\rm{M}}}}}_{0}]+{{{{{{\rm{ln}}}}}}F}({{D}}_{{{{{{\rm{M}}}}}}}-1)$$

where [M_(calc)_] is the calculated concentration of element M and [M_0_] the calculated initial concentration (equivalent to the modelled concentration of the least evolved melt, B11). The problem was solved by minimising χ^2^ defined as:

13 $${{{{{{\rm{\chi }}}}}}}^{2}=\sum {{{\rm{M}}}}\sum {{n}}_{{{{{{\rm{s}}}}}}}{({{{{\mathrm{ln}}}}}[{{{{\rm{M}}}}}_{{{{{{\rm{mes}}}}}}}* {{f}}_{\!{{{\rm{M}}}}}]-{{{{\mathrm{ln}}}}}[{{{{\rm{M}}}}}_{({{{{{\rm{calc}}}}}})}])}^{2}/{{{{{\rm{\sigma }}}}}}{({{{{{{\rm{ln}}}}}}{{{\rm{M}}}}})}^{2}$$

where [M_mes_] are the measured trace element contents of the glasses (Table S2) and σ(lnM)^2^ are taken to be 5% for all the elements except Tl, for which it is 10%. In order to reconcile the data of Sisson et al.^8^ with the new analytical results reported here a correction factor, *f*_M_, was included and refined for each element that was analysed in both this study and Sisson et al.^8^. Due to the large disparity between the Pb contents determined in the two studies this element was excluded from the fit. The value of *F* was set to 1 (i.e., zero crystallisation) for the least evolved sample. While the entire set of glasses form a continuous series in terms of REE the two most evolved glasses (A13 and C4) have anomalous concentrations, relative to the trends defined by the other samples, of some major and trace elements, which are attributed to the presence of an additional fractionating phase (possibly a Fe–Ti oxide). These two glasses were excluded from the fit. For 20 samples and 39 elements, there are 668 observations (allowing for some missing data) and 120 parameters to be refined (*F*, *f*_M_, M_0,_ *D*_M_), giving 548 degrees of freedom. However, the problem is ill-posed due to the high degree of correlation between *F* and *D*_M_, where changes in *F* are compensated by changes in *D*_M_ with insignificant changes to the minimised χ^2^. Nevertheless, the relative magnitudes of the calculated *F* and *D*_M_ remain the same and the most incompatible elements are always Be, La, Ce, Tl, and Th. The model was therefore constrained by setting *D*_Th_ to 0; Th was chosen as it is better determined than Be and Tl, and is independent of the REE modelling. The reduced χ^2^ for the resulting regression is 1.8 indicating that the data are well fit by the model given the assessed uncertainties used in the weighting. The predicted values of *D*_M_ are given in Table S6 and the degree of crystallisation calculated for each glass in Table S4.

### Cotectic assemblage

The REE shape modelling indicates the cotectic crystallisation of garnet and clinopyroxene. To model the cotectic mineral assemblage the bulk cotectic composition needs to be first determined. The good fit of the trace element data to a model of fractional crystallisation suggests that an assumption of constant values of *D*_M_ is not unreasonable. This in turn suggests that the crystallising assemblage did not change significantly during the evolution of the suite. Using this assumption, the values of *F* from the trace element modelling may be used to determine the bulk cotectic composition from a mass balance calculation for each major element oxide component, *y*:

14 $$[{{{{\rm{M}}}}}_{{y}({{{{{\rm{calc}}}}}})}]=([{{{{\rm{M}}}}}_{{y0}}]-[{{{{\rm{M}}}}}_{{y}({{{{{\rm{cotectic}}}}}})}](1-{F}))/{F}$$

where [M_*y*(cotectic)_] is the concentration of *y* in the cotectic composition, [M_*y*0_] the calculated starting composition of the least evolved glass, and [M_*y*(calc)_] the calculated composition of each glass. The problem was solved by minimising χ^2^ defined as:

15 $${{{{{{\rm{\chi }}}}}}}^{2}=\sum {y}\sum {{n}}_{{{{{{\rm{s}}}}}}}{([{{{{\rm{M}}}}}_{{y}({{{{{\rm{mes}}}}}})}]-[{{{{\rm{M}}}}}_{{y}({{{{{\rm{calc}}}}}})}])}^{2}/{{{{{\rm{\sigma }}}}}}{({y})}^{2}$$

where [M_*y*(mes)_] is the measured concentration of each major element oxide component (Table S1), *n*_s_ the sample, and σ(*y*) the uncertainty, which varied according to the uncertainty of the EPMA analyses. The constraint ΣΜ_*y*(cotectic)_ = 100 (wt. %) was imposed during fitting. The bulk cotectic composition determined was 48 wt. % SiO_2_, 1 wt.% TiO_2_, 18 wt. % Al_2_O_3_, 0.2 wt.% MnO, 8 wt.% MgO, 12 wt.% CaO, 2.5 wt.% Na_2_O, 0.5 wt.% K_2_O and 10 wt.% FeO. The reduced χ^2^ for the regression was 1.1.

The bulk composition of the cotectic can then be used to determine the proportions of the cotectic phases. The calculation was carried out in cation fractions, conflating Mg, Fe^2+^, and Mn together as one cation component, since these cations substitute freely for each other in the relevant phases. The composition was decomposed by least-squares regression into the experimentally determined garnet and clinopyroxene compositions (Table S5b) plus ideal end-member phlogopite. The best fit composition was clinopyroxene (63%), garnet (32%), and phlogopite (5%), for which the largest errors were for SiO_2_ (2% excess) and CaO (4% deficit).

## Supplementary information

Supplementary Information

## Data Availability

All data generated or analysed in this study are provided in the Supplementary Information. Source data are provided with this paper.

## Source data

Source Data
