# Supplementary material for: Fractional crystallisation of eclogite during the birth of a Hawaiian Volcano
Source: Nat Commun. 2022 May 26;13:2946. doi: 10.1038/s41467-022-30108-x (PMC9135719; doi:10.1038/s41467-022-30108-x)
Supplement: Supplementary file 1 — Supplementary Information [file 41467_2022_30108_MOESM1_ESM.pdf]

Supplementary Figures

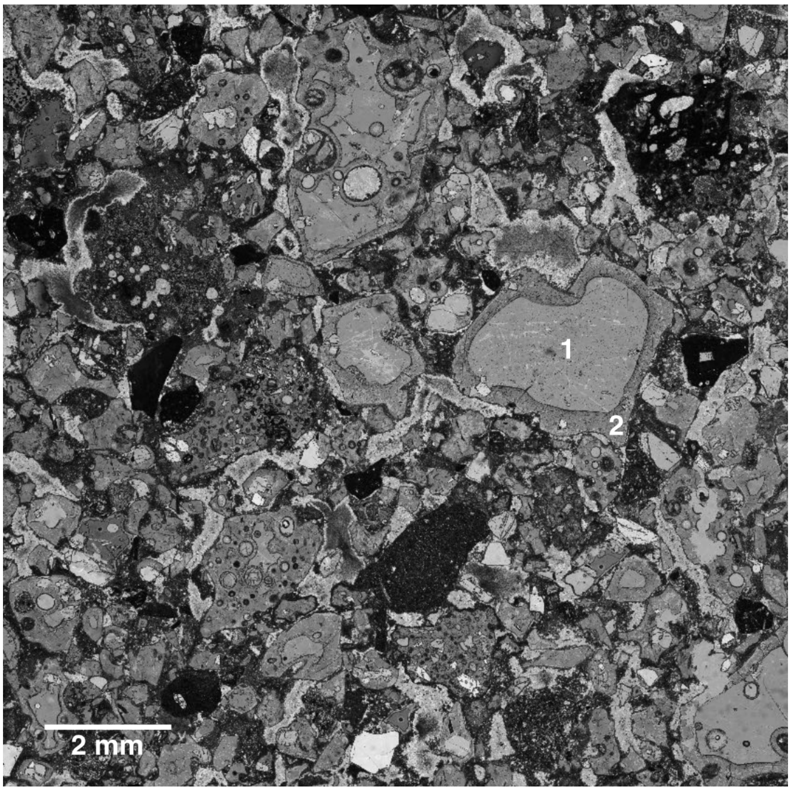

Fig. S1 Optical microscope image (transmitted light) of a thin section from a volcaniclastic sandstone (S508-R3) comprising an assortment of glass grains and lithic particles cemented by palagonite and interstitial zeolites, showing a large glass grain with an unaltered core (1) and palagonatised rim (2).

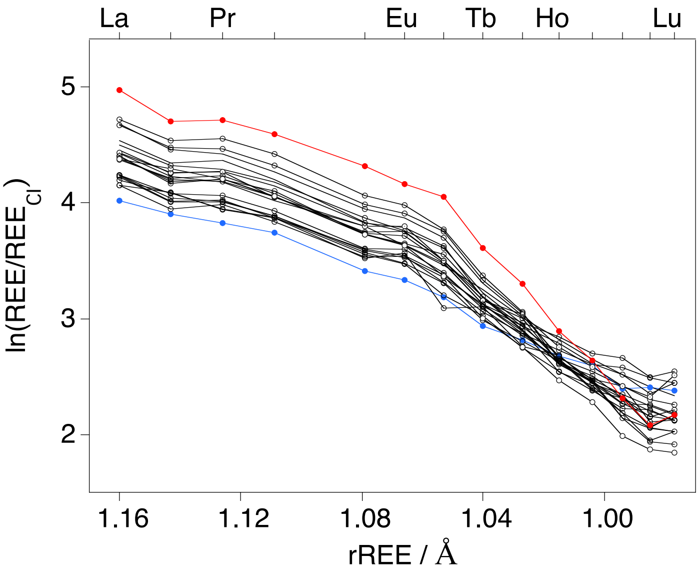

Fig. S2 The chondrite (CI) normalised REE patterns of the basanite-nephelinite suite of glasses, with the most primitive (B11; blue) and evolved (C4; red) samples indicated.

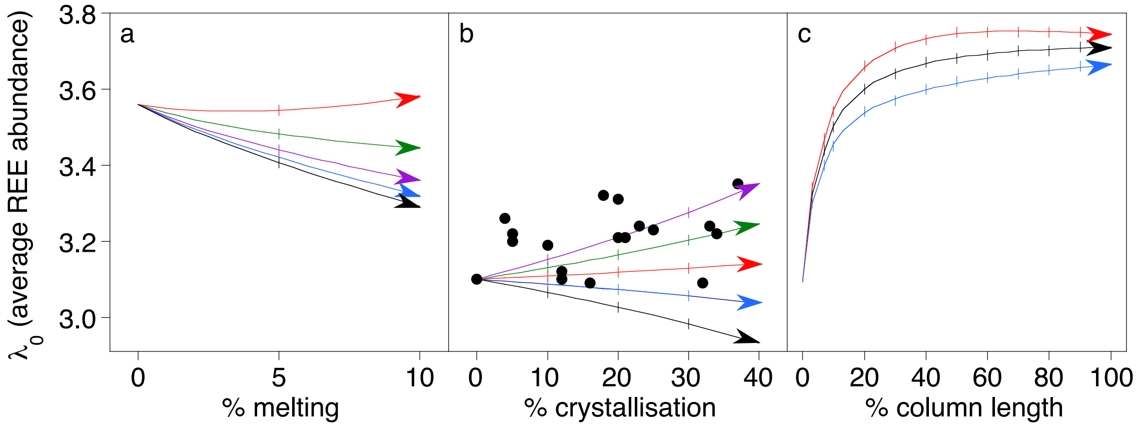

Fig. S3 Trends in λ_0 (average REE abundance) due to **(a)** partial melting starting at the λ_0 of the most evolved sample for 10 % total melting (5 % interval marked) where the relative proportions of clinopyroxene (cpx), orthopyroxene and olivine contributing to the melt are constant but the amount of garnet varies between 2 (black), 7 (blue), 14 (purple), 28 (green) and 50 (red) %, **(b)** fractional crystallisation of 40 % garnet (gt) and cpx (i.e., 60 % melt remaining) from the least evolved sample, where the fractionating assemblage varies from gt:cpx 0.5:0.5 (black), 0.4:0.6 (blue), 0.3:0.7 (red), 0.2:0.8 (green) to 0.1:0.9 (purple), 10 % intervals of crystallisation are marked and the λ_0 of the glasses (black circles; errors similar to or smaller than the size of the symbols) are shown for the calculated degree of crystallisation and **(c)** reactive flow of the least evolved melt flowing through a matrix of garnet peridotite with constant porosity (0.05; black), decreasing porosity (0.05 to 0.01; red) and increasing porosity (0.05 to 0.1; blue), intervals corresponding to flow distances of 3 %, 7 %, 10 % and then 10 % increments to 100 % are marked. Arrows show the directions of the petrogenetic processes. The variation in λ_0 is insensitive to petrogenetic processes compared to λ_1 and λ_2.

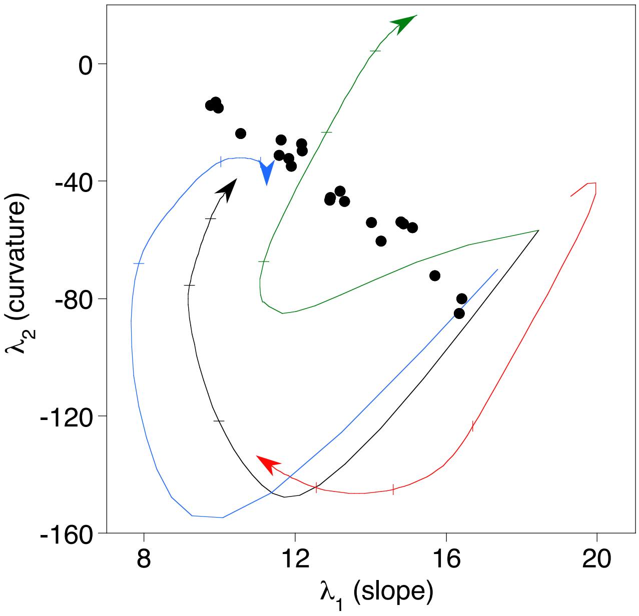

Fig. S4 The λ_2 (curvature) and λ_1 (slope) of the basanite-nephelinite suite (black symbols) and trends in curvature and slope of the REE pattern of melts following the melt front (shown in Fig. 3c) due to reactive flow through a garnet peridotite matrix with constant (0.05; black), decreasing (0.05 to 0.01; red) and increasing (0.05 to 0.1; blue) porosity, and constant porosity while undergoing a mineralogical reaction to pyroxenite (green). The curves start at the points corresponding to the REE pattern of the first batch of melt to pass completely through the column (i.e., at the end of the curves shown in Fig. 3c) and end at points corresponding to the third batch of melt to pass through the column, with the intervals indicating the REE pattern of melt exiting the column 1.5, 2.0, and 2.5 column lengths behind the melt front. Arrows show the direction of the petrogenetic processes.

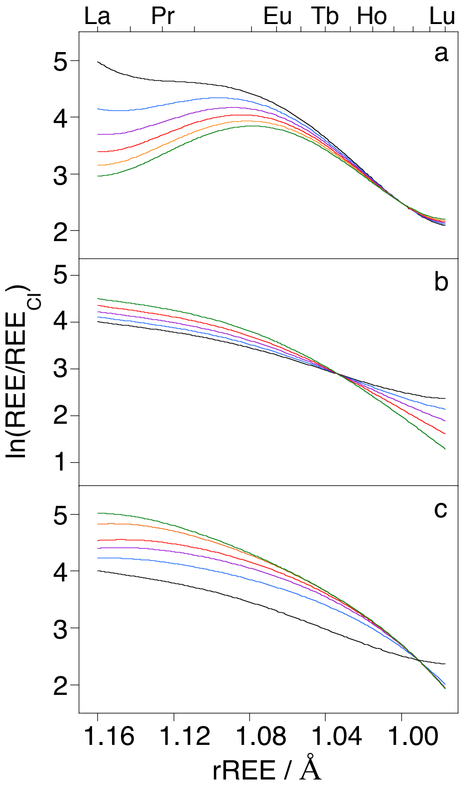

Fig. S5 Modelled REE patterns due to **(a)** 2% (blue), 4% (purple), 6% (red), 8% (orange) and 10% (green) partial melting, starting with the REE pattern of the most evolved melt (C4; black), where the proportion of garnet contributing to the melt is 2 %; these REE patterns correspond to slope and curvature loci at 2, 4, 6, 8 and 10 % melting on the red vector in Fig. 3a, **(b)** 10% (i.e., 90 % melt remaining; blue), 20% (purple), 30% (red) and 40% (green) fractional crystallisation from the least evolved sample (B11; black) of an assemblage comprising garnet (30 %) and clinopyroxene (70 %); these REE patterns correspond to slope and curvature loci at 10, 20, 30 and 40 % crystallisation on the red vector in Fig. 3e, and **(c)** reactive melt flow, where the initial melt (B11; black) has travelled 3 % (blue), 7 % (purple), 10 % (red), 20 % (orange) and 30 % (green) of the column through which it is flowing; these REE patterns correspond to slope and curvature loci at 3, 7, 10, 20 and 30 % total flow distance on the black vector in Fig. 3c.

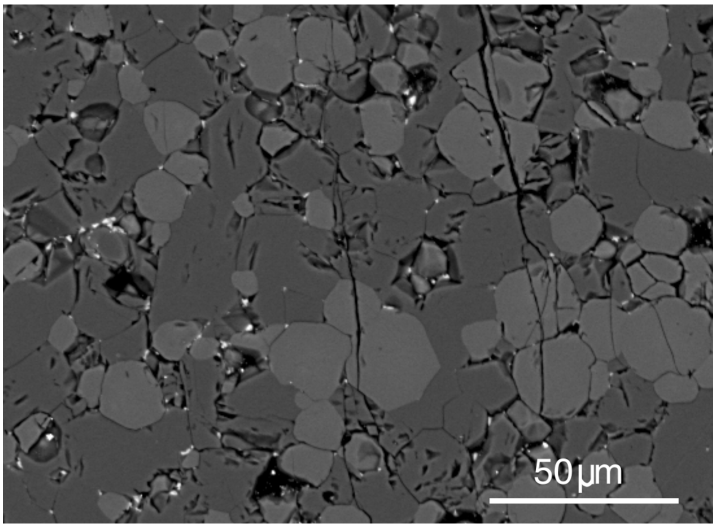

Fig. S6 Back scattered electron image of garnet (light grey), clinopyroxene (dark grey) and minor hercynite (white spots) in equilibrium with melt (not visible) at 5 GPa and 1450 ˚C.

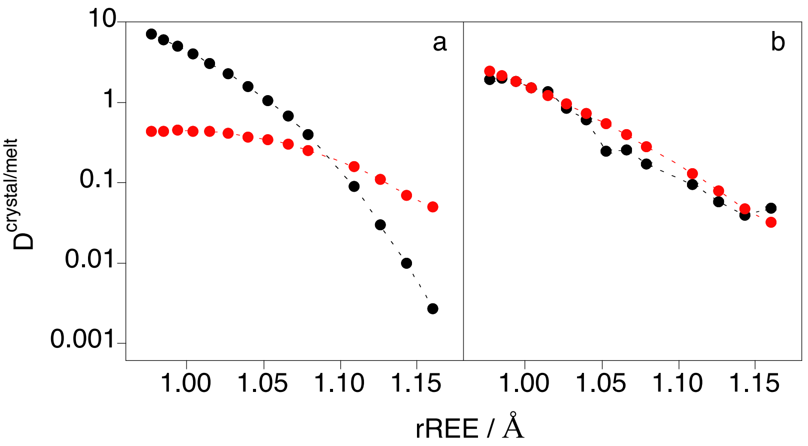

Fig. S7 (a) Garnet (black) and clinopyroxene (red) REE crystal-melt partition coefficients (from O'Neill 2016) used for modelling the petrogenetic process vectors (shown in Fig. 3) and **(b)** the calculated bulk REE partition coefficients for the suite of glasses (black; Table S6) and those obtained from summing the garnet and clinopyroxene partition coefficients shown in (a) in the proportions of the modelled fractionating assemblage (32% garnet and 63% clinopyroxene; red).

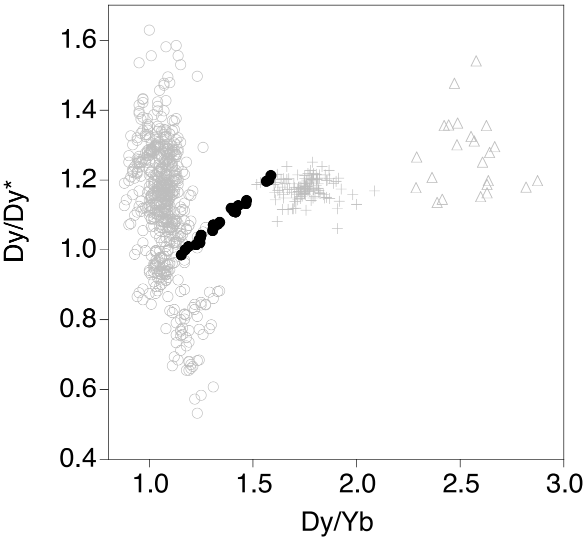

Fig. S8 Dy/Dy* and Dy/Yb of ocean floor basalts[10] (open circles), Hawaiian shield tholeiites[13] (crosses), melts from Loihi[14] (triangles) and the pre-shield Kilauean basanite-nephelinite glasses of this study (black). The pre-shield glasses fall on the garnet fractional crystallisation vector modelled for Dy/Dy* and Dy/Yb (see Fig. 4d in Davidson et al.[26]). Dy was not determined for the Loihi melts for which garnet fractional crystallisation has been suggested (red and green triangles in Fig. 1)

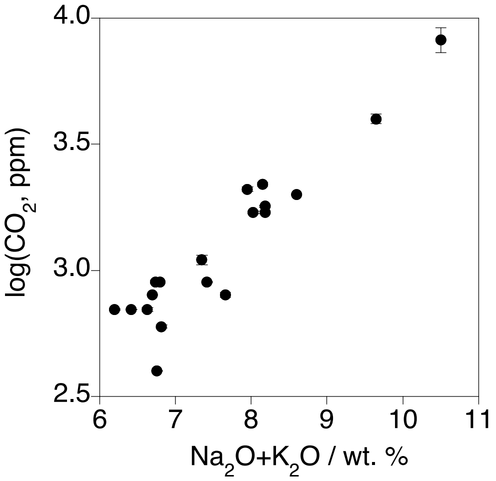

Fig. S9 CO_2 contents of the basanite-nephelinite suite of glasses versus the total alkali content.

Supplementary Tables

Table S1 Major element concentrations of the basanite-nephelinite glasses determined by electron probe microanalysis and H_2O and CO_2 contents determined by ATR-FTIR spectroscopy (wt.%). The uncertainty is one standard deviation. Sample nomenclature is from Sisson et al.[8]. The samples are ordered according to their total alkali content from low (B11) to high (C4), as in Sisson et al.[8].

Sample	SiO_2	TiO_2	Al_2O_3	FeO	MnO	MgO	CaO	Na_2O	K_2O	P_2O_5	SO_3	Cl	H_2O	CO_2	Total
B11	41.74(6)	2.24(4)	14.90(10)	11.54(9)	0.20(1)	6.65(4)	12.69(4)	4.78(8)	1.51(3)	0.35(1)	0.29(2)	0.08(1)	0.69(1)	0.068(3)	97.8(2)
C22	42.61(2)	2.23(4)	15.10(10)	11.59(6)	0.18(2)	6.45(4)	12.73(3)	4.87(7)	1.50(1)	0.37(1)	0.32(1)	0.10(1)	0.73(1)	0.070(1)	98.9(2)
C1	41.62(9)	2.34(3)	14.72(2)	11.86(8)	0.19(2)	6.52(8)	12.72(3)	4.80(30)	1.50(1)	0.34(1)	0.34(1)	0.11(1)	0.736(4)	0.070(6)	97.9(2)
A2	42.30(6)	2.28(1)	15.43(2)	11.58(6)	0.20(1)	6.57(7)	12.74(3)	5.23(5)	1.56(2)	0.41(1)	0.34(3)	0.11(1)	0.70(1)	0.079(3)	99.5(2)
A4	41.90(20)	2.35(2)	14.85(4)	12.03(7)	0.18(2)	6.66(2)	12.95(4)	5.30(30)	1.53(4)	0.39(1)	0.37(3)	0.11(1)	0.63(1)	0.091(6)	99.3(2)
C13	42.14(4)	2.68(3)	14.87(4)	12.68(6)	0.17(3)	6.53(6)	12.76(9)	3.30(40)	1.68(2)	0.37(1)	0.39(1)	0.11(1)	0.82(1)	0.042(1)	98.6(2)
B10	41.10(10)	2.34(3)	14.50(10)	11.80(10)	0.18(1)	6.60(20)	13.20(10)	5.20(20)	1.56(1)	0.40(3)	0.37(3)	0.11(1)	0.69(1)	0.087(3)	98.5(2)
B19	41.10(20)	2.61(2)	14.40(10)	12.33(1)	0.19(1)	6.53(4)	12.69(3)	5.29(2)	1.69(1)	0.40(1)	0.41(1)	0.11(1)	0.82(1)	0.063(7)	98.6(2)
C12*	41.9	2.37	14.9	12.2	0.19	6.52	13.2	5.32	1.53	0.41	0.355	0.097	0.76(4)	-	-
C6	40.80(20)	2.74(2)	14.35(7)	12.70(10)	0.17(2)	6.29(4)	12.90(7)	5.78(2)	1.81(2)	0.42(2)	0.46(1)	0.11(1)	0.82(3)	0.11(2)	99.6(2)
C18	40.90(40)	2.75(2)	14.10(10)	13.00(30)	0.19(3)	6.40(10)	13.10(20)	5.40(40)	1.77(1)	0.45(3)	0.45(2)	0.11(1)	0.91(3)	0.094(3)	99.8(2)
A22	40.53(6)	2.40(3)	14.54(3)	12.05(2)	0.20(2)	6.34(4)	13.63(1)	6.16(8)	1.64(1)	0.50(1)	0.46(1)	0.13(1)	-	-	98.6(2)
A1	40.40(10)	2.64(2)	14.34(3)	12.57(4)	0.18(1)	6.30(10)	13.19(2)	5.94(7)	1.76(1)	0.45(1)	0.56(2)	0.12(1)	0.73(1)	0.079(9)	99.4(2)
A27	40.29(5)	2.75(2)	14.18(5)	12.60(10)	0.20(1)	6.15(7)	13.10(2)	6.06(5)	1.84(1)	0.46(4)	0.48(1)	0.13(1)	-	-	98.3(2)
A17	39.61(5)	2.87(2)	13.90(10)	13.20(10)	0.17(2)	5.97(7)	13.30(10)	6.06(2)	1.95(2)	0.49(2)	0.57(1)	0.14(1)	0.97(2)	0.21(1)	99.6(2)
B20	39.17(4)	2.86(1)	13.67(8)	13.15(1)	0.20(1)	6.17(2)	12.97(7)	6.26(7)	1.93(2)	0.50(2)	0.52(1)	0.13(1)	0.90(2)	0.173(6)	98.6(2)
A7	39.76(6)	2.83(1)	13.98(5)	12.92(1)	0.16(1)	6.08(4)	13.30(3)	6.29(2)	1.92(1)	0.53(2)	0.61(1)	0.15(1)	0.91(1)	0.222(7)	99.7(2)
C23	40.40(80)	2.90(7)	13.90(20)	13.13(8)	0.19(1)	6.10(10)	13.60(40)	6.40(40)	2.07(4)	0.57(2)	0.56(5)	0.14(1)	1.00(2)	0.172(6)	101.3(2)
C3	40.15(7)	2.66(1)	14.00(4)	12.72(7)	0.20(1)	6.25(1)	12.92(3)	5.40(10)	1.79(2)	0.42(5)	0.48(1)	0.13(1)	0.84(1)	0.183(4)	98.2(2)
C20	39.90(60)	3.16(6)	13.70(20)	13.20(10)	0.18(3)	5.97(7)	13.30(20)	5.20(20)	2.30(8)	0.60(2)	0.59(3)	0.15(1)	1.08(3)	0.204(3)	99.7(2)
A13	39.00(40)	2.92(5)	13.52(6)	11.90(10)	0.17(2)	5.50(8)	13.90(10)	7.50(10)	2.31(5)	0.63(1)	0.72(4)	0.17(1)	0.99(2)	0.40(2)	99.0(2)
C4	37.40(20)	2.85(3)	12.80(9)	11.60(20)	0.20(2)	5.54(6)	13.76(6)	8.27(7)	2.52(7)	0.78(1)	0.80(1)	0.19(1)	1.04(4)	0.82(5)	98.7(2)

-, not analysed

* not analysed, values Sisson et al.[8] except H_2O

Table S2 Trace element concentrations (ppm) of the basanite-nephelinite glasses determined by LA-ICPMS; 1# and 2# are the number of analyses for routine 1 and routine 2. The uncertainty is one standard deviation and is given for analyses from this study, other than for B19 and C1 for which only one analysis was possible. All other data (i.e., those without errors) are from Sisson et al.[8]. The samples are ordered as in Table S1.

Sample	1#	2#	Li	Be	Sc	V	Cr	Mn	Co	Ni	Cu	Zn	Ga	Rb
B11	3	2	8.0(2)	1.42(1)	31.9(2)	338(1)	129(1)	1582(15)	47.9(3)	72.4(19)	91(1)	123(2)	22.4(1)	32.3(7)
C22	0	2	8.3(2)	1.50(5)	32.6(4)	348(1)	129(1)	1636(8)	48.9(5)	69.7(3)	93(1)	130(1)	22.8(1)	28.6
C1	0	1	8.2	1.37	29.2	357	120	1646	51.6	75.2	97.7	140	24.4	27.6
A2	0	3	9.0(5)	1.60(7)	32.3(2)	327(4)	114(2)	1607(38)	47.1(13)	68.2(30)	88(1)	138(6)	22.2(3)	31.0
A4	0	2	8.7(3)	1.52(6)	29.4(2)	357(1)	111(5)	1624(5)	50.6(3)	71.8(21)	93(1)	149(1)	23.4(2)	31.0
C13	0	0	-	-	-	-	-	-	-	-	-	-	-	33.0
B10	3	0	-	-	-	-	-	-	-	-	-	-	-	30(1)
B19	1	0	-	-	-	-	-	-	-	-	-	-	-	38.8
C12	0	0	-	-	-	-	-	-	-	-	-	-	-	30.2
C6	0	2	9.20(2)	1.75(7)	23.9(1)	372(4)	76(8)	1575(14)	50.7(6)	61.6(4)	89(1)	166(1)	26.7(6)	33.6
C18	0	0	-	-	-	-	-	-	-	-	-	-	-	33.9
A22	0	2	13.5(1)	1.90(9)	26.1(1)	361(1)	75(2)	1893(13)	53.9(1)	65.7(15)	105(1)	121(1)	26.8(5)	30.1
A1	0	3	9.5(9)	1.9(1)	23.9(2)	361(16)	69(4)	1577(180)	49.2(55)	56.0(60)	86(10)	159(9)	25(3)	33.3
A27	0	3	10.7(1)	1.94(7)	22.5(1)	369(3)	52(1)	1657(8)	50.8(1)	59.6(13)	91(1)	152(6)	28.2(3)	33.1
A17	0	0	-	-	-	-	-	-	-	-	-	-	-	35.4
B20	3	3	11.22(3)	1.83(4)	21.2(1)	370(2)	35(1)	1560(10)	48.7(8)	50.7(26)	84(1)	179(4)	27.4(1)	40.7(6)
A7	0	2	11.0(1)	2.21(1)	22.4(2)	367(1)	25(1)	1571(17)	48.1(1)	46.9(5)	80(2)	187(1)	26.5(7)	37.2
C23	0	0	-	-	-	-	-	-	-	-	-	-	-	37.3
C3	0	2	10.6(2)	1.96(10)	21.6(4)	364(4)	30(1)	1559(29)	49.1(6)	52(2)	86(2)	179(6)	27.0(7)	34.8
C20	0	0	-	-	-	-	-	-	-	-	-	-	-	40.0
A13	0	2	13.6(1)	2.62(1)	18.1(2)	347(5)	0.1(1)	1528(24)	40.5(16)	18.4(26)	79(1)	200(5)	29(1)	41.7
C4	0	2	16.6(1)	2.91(5)	16.9(1)	333(1)	0.09(2)	1569(6)	38.6(1)	15.0(5)	78(1)	213(5)	28.1(2)	40.1

-, not analysed

Table S2 cont.

Sample	Sr	Y	Zr	Nb	Mo	Sn	Ba	La	Ce	Pr	Nd	Sm	Eu	Gd
B11	561(6)	19.8(6)	130(1)	17.6(3)	0.85(4)	2.11(3)	412(3)	13.7(3)	31.2(4)	4.36(5)	20.2(3)	4.66(12)	1.66(7)	4.97(4)
C22	664	26.7	148	20.6	0.97(1)	2.26(4)	443	17.1	35.8	5.23	23.3	5.57	2.01	5.91
C1	675	24.2	145	20.7	1.01	2.31	429	17.0	34.8	5.31	23.0	5.63	2.05	6.02
A2	669	25.0	139	20.7	1.00(6)	2.15(10)	446	15.7	32.7	5.12	22.2	5.26	2.04	5.63
A4	705	22.6	139	21.9	1.00(1)	2.31(2)	480	17.1	37.2	5.51	24.4	5.67	2.16	6.13
C13	673	21.5	156	21.9	-	-	484	16.8	34.6	5.21	23.3	5.48	1.92	5.60
B10	681(18)	19.2(8)	127(5)	19.4(17)	-	-	451(16)	15.7(12)	37.6(10)	4.87(47)	22.9(26)	5.20(55)	2.09(23)	4.53(86)
B19	660	18.9	148	19.7	-	-	478	16.4	37.4	4.92	23.1	5.40	1.91	5.05
C12	773	26.2	154	23.2	-	-	536	20.7	43.1	6.19	26.6	6.89	2.23	6.55
C6	786	21.4	162	24.4	1.19(7)	2.59(9)	544	19.8	42.0	6.22	27.6	6.42	2.25	6.67
C18	818	22.9	160	23.8	-	-	529	20.1	41.3	6.35	28.9	6.41	2.28	7.18
A22	826	22.9	138	24.4	1.49(4)	3.28(13)	528	20.8	44.7	6.74	27.4	6.88	2.63	6.82
A1	799	21.2	154	24.6	1.30(35)	2.42(41)	537	19.5	42.4	6.54	28.2	6.46	2.43	6.70
A27	805	21.3	164	24.5	1.69(7)	2.62(6)	517	20.2	40.6	6.37	27.6	6.54	2.52	6.82
A17	874	21.0	176	27.1	-	-	594	23.1	48.5	7.46	31.9	7.10	2.51	6.79
B20	847(3)	16.4(2)	155(1)	24.4(4)	1.28(3)	2.47(6)	566(7)	19.7(1)	46.0(2)	6.38(22)	27.6(2)	6.39(17)	2.23(10)	5.96(9)
A7	878	20.6	175	26.3	1.20(4)	2.63(7)	592	21.3	44.5	6.79	30.2	7.06	2.61	7.75
C23	1018	22.6	177	29.5	-	-	648	26.6	54.6	7.90	34.1	7.98	2.84	8.30
C3	881	21.0	172	26.5	1.33(9)	2.51(4)	566	22.2	47.4	6.90	31.2	7.36	2.53	7.63
C20	1050	21.3	202	31.2	-	-	661	26.3	55.4	8.25	36.1	8.30	2.94	8.75
A13	1061	21.7	206	34.4	1.66(6)	2.69(1)	703	27.7	58.9	9.01	39.8	8.97	3.17	8.91
C4	1363	27.0	252	41.2	2.11(17)	2.95(8)	794	35.6	69.5	10.55	47.3	11.55	3.80	11.79

-, not analysed

Table S2 cont.

Sample	Tb	Dy	Ho	Er	Tm	Yb	Lu	Hf	Ta	Tl	Pb	Th	U
B11	0.70(1)	4.25(8)	0.81(2)	2.20(16)	0.28(1)	1.87(2)	0.27(1)	3.42(12)	0.93(4)	0.048(4)	2.65(4)	1.02(1)	0.29(1)
C22	0.85	4.91	0.95	2.45	0.37	2.04	0.32	3.71	1.28	0.054(6)	1.01	1.40	0.27
C1	0.84	4.78	0.90	2.24	0.32	1.77	0.29	3.10	1.24	0.041	1.05	1.31	0.27
A2	0.80	4.48	0.85	2.23	0.34	2.03	0.29	3.19	1.16	0.050(5)	1.17	1.18	0.28
A4	0.82	4.41	0.79	2.10	0.29	1.68	0.24	3.19	1.24	0.051(4)	1.26	1.27	0.35
C13	0.74	4.19	0.77	1.96	0.29	1.39	0.21	3.78	1.25	-	1.23	1.29	0.28
B10	0.84(11)	4.02(68)	0.81(5)	1.89(21)	0.25(3)	1.61(9)	0.22(4)	3.18(16)	0.97(11)	-	3.05(11)	1.31(16)	0.37(3)
B19	0.77	3.97	0.70	1.77	0.24	1.54	0.21	3.78	1.06	-	3.70	1.20	0.35
C12	0.97	5.14	0.93	2.33	0.32	1.88	0.26	3.79	1.38	-	1.34	1.67	0.32
C6	0.85	4.48	0.78	1.79	0.26	1.52	0.21	4.04	1.42	0.054(3)	1.44	1.61	0.32
C18	0.91	4.77	0.81	1.89	0.27	1.59	0.22	3.74	1.37	-	1.47	1.71	0.34
A22	0.88	5.30	0.87	2.18	0.29	1.72	0.31	3.75	1.45	0.082(11)	1.38	1.54	0.37
A1	0.85	4.59	0.76	1.93	0.25	1.46	0.21	3.66	1.40	0.075(18)	1.72	1.45	0.34
A27	0.88	5.12	0.76	2.02	0.26	1.45	0.23	4.31	1.58	0.086(4)	1.35	1.47	0.31
A17	0.91	4.63	0.75	1.91	0.22	1.31	0.19	4.53	1.58	-	1.57	1.68	0.39
B20	0.76(2)	3.99(5)	0.65(2)	1.61(10)	0.19(1)	1.10(2)	0.159(4)	3.85(17)	1.24(3)	0.068(7)	3.93(18)	1.41(2)	0.43(2)
A7	0.90	4.60	0.70	1.86	0.23	1.32	0.19	4.06	1.50	0.088(4)	1.67	1.54	0.38
C23	1.02	5.23	0.80	1.95	0.27	1.32	0.22	4.16	1.71	-	1.70	2.15	0.41
C3	0.94	4.88	0.77	1.90	0.26	1.33	0.22	4.23	1.59	0.082(4)	1.54	1.79	0.36
C20	1.05	5.34	0.79	1.81	0.23	1.18	0.19	4.74	1.96	-	1.69	2.07	0.39
A13	1.09	5.40	0.75	1.94	0.22	1.17	0.17	4.59	2.02	0.102(1)	2.03	2.07	0.47
C4	1.38	6.89	1.00	2.30	0.26	1.35	0.22	5.97	2.47	0.107(2)	2.10	2.86	0.49

-, not analysed

Table S3 Some common canonical trace-element (ppm) ratios, for the least evolved (C22) and most evolved (C20) glasses analysed in the same LA-ICPMS session (excluding A13 and C4 due to their crystallisation of an Fe-Ti oxide phase; see Methods), the bulk silicate Earth (BSE[1]), ocean floor basaltic glasses (OFB[2, 3]), and the bulk continental crust (CC[4]).

	Least evolved (C22)	Most evolved (C20)	BSE	OFB[a]	CC
Large ion lithophile elements					
K/U (x 10^{-4})	4.7	4.7	1.1	1.4(4)*	1.15
Ba/Rb	15.5	16.5	11.3	10.8(14)	9.3
Ba/Th	316	319	81	79(25)	81
Sr/Nd	28	29	16.4	13(4)*	16
High field strength elements					
(Sm+Gd)/Ti (x 10^{4})	6.7	7.2	8.1	8.6(5)	18
Hf/Sm	0.67	0.57	0.69	0.70(6)	0.95
Ta/Th	0.91	0.94	0.51	0.92(18)	0.13
Zr/Hf	40	42	34	37(3)*	36
Nb/Ta	16	16	14	16.8(12)	11
Th/U	5.2	5.3	3.9	2.95(66)	4.3
Volatiles					
H_2O/Ce	203	195	616	250(111)[b]	-
Cl/La	46	49	44	16[c]	12
Others					
Ca/Al	1.11(1)	1.30(3)	1.10	1.02(9)	0.54
Na/Ti	1.03(2)	1.00(3)	2.0	2.4(6)*	2.0
Fe/Mn	60(5)	62(5)	60	53(2)	67

[a] > 5.5 wt% MgO only
[b] mode = 212
[c] mode
* these ratios vary systematically with MgO

Table S4 The λ_n coefficients determined from fits of the f_n orthogonal polynomials to the natural logarithm chondrite normalised REE patterns of each sample (Eq. 1), and the degree of crystallisation (1-F) determined from the fit of Eq. 10 to the trace element data. The uncertainty on F is < 0.01 for a 5 % uncertainty on the trace element data.

Sample	Average abundance λ_0	Slope λ_1	Curvature λ_2	Degree of crystallisation (1-F)
B11	3.09(2)	9.8(3)	-14(6)	0.00
C22	3.36(2)	9.9(3)	-13(6)	0.04
C1	3.22(2)	10.6(3)	-24(7)	0.05
A2	3.19(2)	10.0(4)	-15(7)	0.05
A4	3.18(2)	11.6(3)	-31(7)	0.10
C13	3.11(2)	11.9(3)	-35(6)	0.12
B10	3.10(2)	11.6(3)	-26(7)	0.12
B19	3.08(2)	12.2(3)	-30(7)	0.15
C12	3.31(2)	11.8(3)	-32(5)	0.18
C6	3.20(2)	13.2(4)	-44(8)	0.20
C18	3.24(3)	12.9(4)	-47(9)	0.23
A22	3.31(3)	12.2(5)	-27(11)	0.20
A1	3.21(2)	13.3(4)	-47(8)	0.22
A27	3.23(3)	12.9(4)	-46(9)	0.25
A17	3.24(2)	14.8(4)	-54(8)	0.33
B20	3.09(2)	15.1(4)	-56(7)	0.32
A7	3.22(3)	14.3(5)	-60(11)	0.34
C23	3.34(3)	14.9(5)	-55(10)	0.37
C3	3.27(3)	14.8(5)	-54(10)	0.32
C20	3.32(3)	15.7(5)	-72(11)	0.41
A13	3.35(3)	16.4(5)	-80(10)	-
C4	3.56(3)	16.3(6)	-85(12)	-

Table S5a Experimental conditions and products of piston-cylinder experiments

Experiment	Pressure (GPa)	Temperature (˚C)	Products
D2528	3.0	1300	cpx, hcn, melt
D2453	4.0	1350	cpx, melt
UHPPC 404	5.0	1450	cpx, gt, hcn, melt

cpx = clinopyroxene; hcn = hercynite; gt = garnet.

Table S5b Major element concentrations (wt. %) of garnet and clinopyroxene synthesised at 5 GPa and 1450 ˚C determined by EDS, normalised to 100 %. The uncertainty is one standard deviation.

Crystal	SiO_2	TiO_2	Al_2O_3	FeO	MnO	MgO	CaO	Na_2O	K_2O
Garnet	40.2(1)	1.27(9)	22.1(1)	11.5(1)	0.31(5)	9.8(1)	14.4(1)	0.20(5)	0.02(1)
Clinopyroxene	49.4(1)	0.91(7)	16.25(5)	4.71(5)	0.06(2)	8.60(4)	15.8(1)	4.12(9)	0.05(1)

Table S6 Bulk crystal-melt partition coefficients (D_M) predicted from fitting the trace element content of the glasses to the general equation for fractional crystallisation (Eq. 10). The error is ~5% of the value.

Element	D_M
Compatible elements	
Cr	4.96
Sc	2.08
Yb	2.01
Ni	2.01
Lu	1.92
Tm	1.83
Er	1.50
Y	1.37
Ho	1.37
Cu	1.26
Mn	1.06
Moderately compatible elements	
Co	0.99
Dy	0.85
V	0.79
Tb	0.60
H_2O	0.57
Sn	0.53
Ga	0.50
Zr	0.39
Rb	0.39
Hf	0.37
Eu	0.25
Gd	0.24
Li	0.20
Nb	0.20
U	0.20
Ba	0.18
Ta	0.17
Zn	0.17
Sm	0.17
Incompatible elements	
Nd	0.09
P	0.08
Be	0.06
Sr	0.06
Pr	0.06
La	0.05
Ce	0.04
Mo	<0.01
Tl	<0.01

References

1 Palme, H. and O'Neill, H.St.C. 2014. Cosmochemical estimates of mantle composition. In: Holland H.D. and Turekian, K.K. (Editors), Treatise on Geochemistry 2nd Edition, Volume 3, *Elsevier,* 1-39.

2 Jenner, F.E. and O'Neill, H.St.C. 2012. Analysis of 60 elements in 616 ocean floor basaltic glasses. Geochemistry, Geophysics, Geosystems, **13**, GC004009.

3 Le Voyer, M., Cottrell, E., Kelley, K.A., Brounce, M. and Hauri, E.H. 2015. The effect of primary versus secondary processes on the volatile content of MORB glasses: An example from the equatorial Mid-Atlantic Ridge (5° N–3° S). *Journal of Geophysical Research: Solid Earth*, **120**, 125-14

4 Rudnick, R.L., Gao, S. 2003. Composition of the continental crust. In: Holland, H. D. & Turekian, K. K. (eds) *Treatise on Geochemistry*, Vol. 3, 2nd edition. Elsevier, 1-64.
